# Supplementary material for: Online dynamical learning and sequence memory with neuromorphic nanowire networks
Source: Nat Commun. 2023 Nov 1;14:6697. doi: 10.1038/s41467-023-42470-5 (PMC10620219; doi:10.1038/s41467-023-42470-5)
Supplement: Supplementary file 1 — Supplementary Information [file 41467_2023_42470_MOESM1_ESM.pdf]

Online dynamical learning and sequence memory with neuromorphic nanowire networks - Supplementary Information

Ruomin Zhu[1*†], Sam Lilak[2†], Alon Loeffler[1], Joseph Lizier[3,4], Adam Stieg[5,6*], James Gimzewski[2,5,6,7*] and Zdenka Kuncic[1,4,8*]

[1]School of Physics, The University of Sydney, Sydney, NSW, Australia.
[2]Department of Chemistry and Biochemistry, University of California, Los Angeles, Los Angeles, CA, United States.
[3]School of Computer Science, The University of Sydney, Sydney, NSW, Australia.
[4]Centre for Complex Systems, The University of Sydney, Sydney, NSW, Australia.
[5]California NanoSystems Institute, University of California, Los Angeles, Los Angeles, CA, United States.
[6]WPI Center for Materials Nanoarchitectonics (MANA), National Institute for Materials Science (NIMS), Tsukuba, Japan.
[7]Research Center for Neuromorphic AI Hardware, Kyutech, Kitakyushu, Japan.
[8]The University of Sydney Nano Institute, Sydney, NSW, Australia.

*Corresponding author(s). E-mail(s): rzhu0837@sydney.edu.au; stieg@cnsi.ucla.edu; gim@chem.ucla.edu; zdenka.kuncic@sydney.edu.au;
†These authors contributed equally to this work.

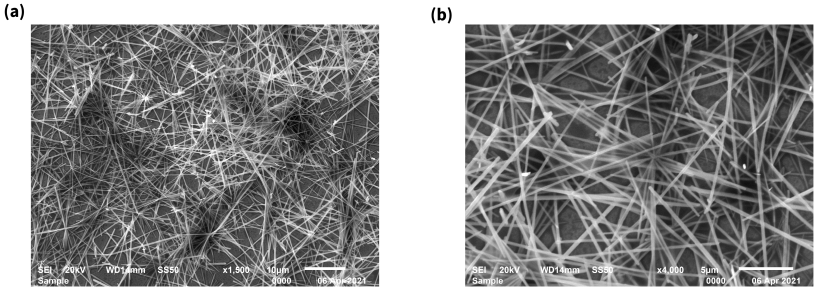

Supplementary Figure S1. SEM images of the nanowire network without electrodes. Scale bars are: (a) $10\,\mu$m; and (b) $5\,\mu$m.

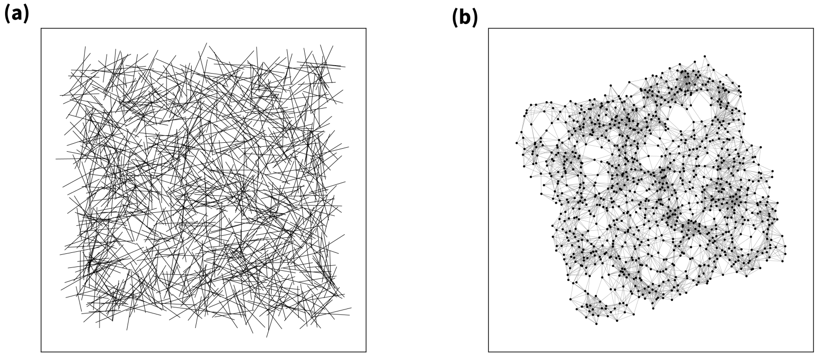

Supplementary Figure S2. Simulated nanowire network. (a) Network of 1024 nanowires with centres and lengths drawn from a uniform and Gamma distribution, respectively. (b) Corresponding graph representation, with nanowires represented by nodes and memristive cross-points between nanowires represented by edges (6877 total edges). Simulations are based on a physically-motivated model from previous studies [1–4].

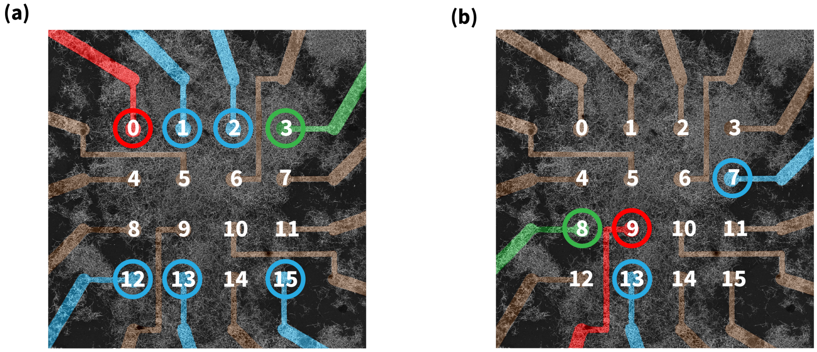

Supplementary Figure S3. Different device electrode setups. (a) Using electrode 0 as input (red) and electrode 3 as drain (green), while reading out from electrodes 1, 2, 12, 13 and 15 (blue). This is the setup used for the online MNIST classification task presented in main Results. (b) An alternate setup using electrode 9 as input and electrode 8 as drain, with readouts from electrodes 7 and 13. This is the setup used for the online image reconstruction task presented in main Results. During experiments, the voltage of every electrode and the drain current are measured continuously.

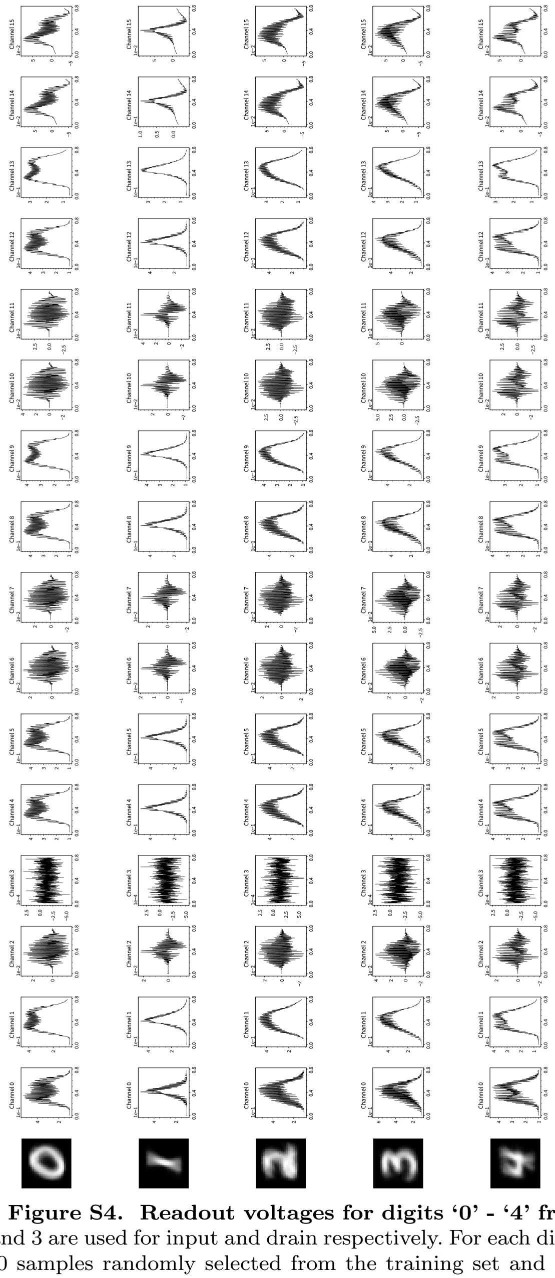

Supplementary Figure S4. Readout voltages for digits '0' - '4' from all 16 channels. Channel 0 and 3 are used for input and drain respectively. For each digit class, readouts correspond to 100 samples randomly selected from the training set and averaged for each channel.

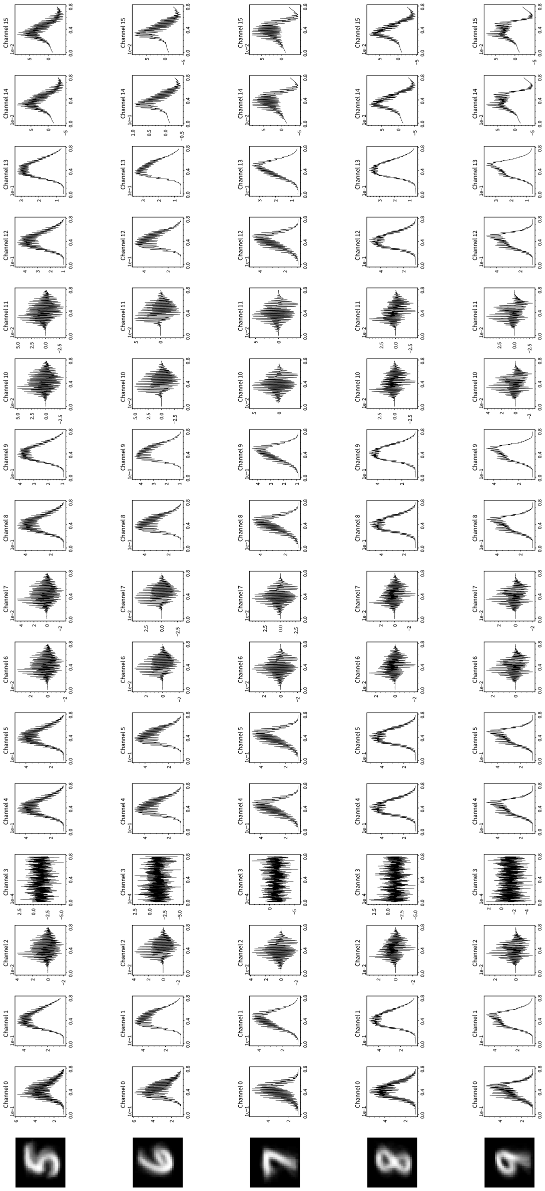

Supplementary Figure S5. Readout voltages for digits '5' - '9' from all 16 channels. Channel 0 and 3 are used for input and drain respectively. For each digit class, readouts correspond to 100 samples randomly selected from the training set and averaged for each channel.

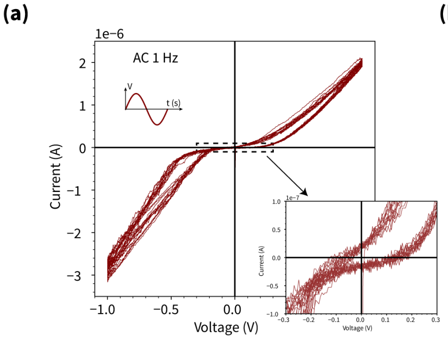

Supplementary Figure S6. $I - V$ **curves under AC sweeps.** (a) Response to 1 Hz sine wave. Inset is a zoom-in near the origin, showing a non-zero crossing. (b) Response to 10 Hz sine wave, showing curves driven further away from zero. See Supplementary Note 1 for further information.

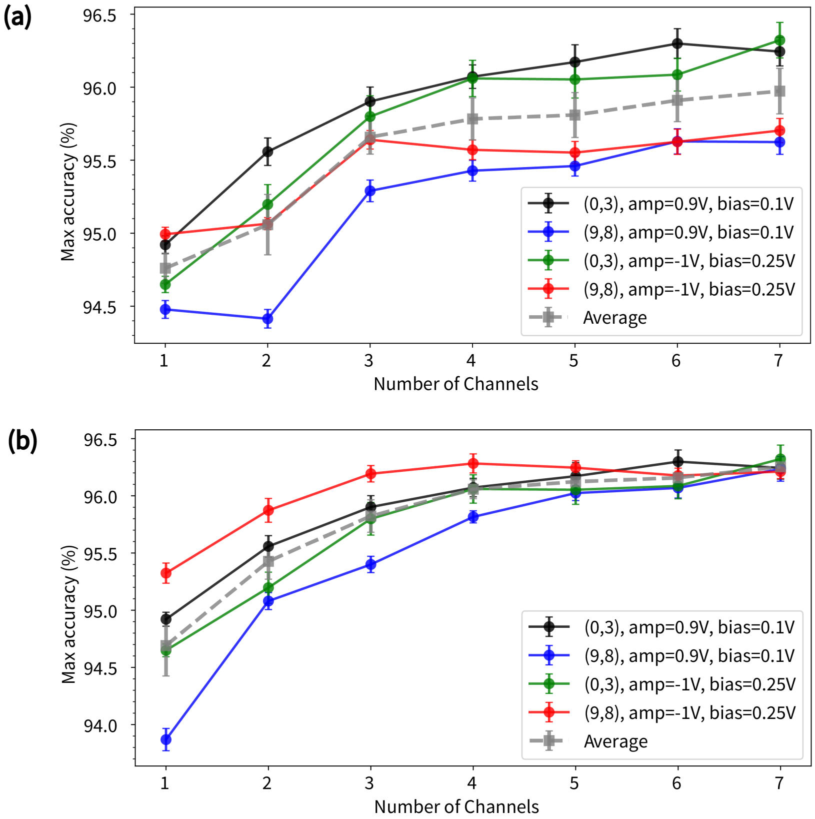

Supplementary Figure S7. MNIST online classification accuracy as a function of number of channels using different electrodes for (input,drain) and different voltage amplitdue and bias. Maximum accuracy for 1,000 testing samples (after training on 50,000 samples) with respect to number of readout channels used by the online linear classifier. Error bars indicate the standard error of the mean of 5 measurements with different sequences of the training samples. (a) Source/drain electrodes (0,3), with channels 1,2,13,15,12,9,14 used for readout to the classifier; and source/drain electrodes (9,8), with channels 1,0,13,3,12,6,15 used for readout. (b) Same as (a), but with channels 12,7,14,1,0,13,3 used for (9,8). For all cases, readouts are streamed to the classifier in the specified order.

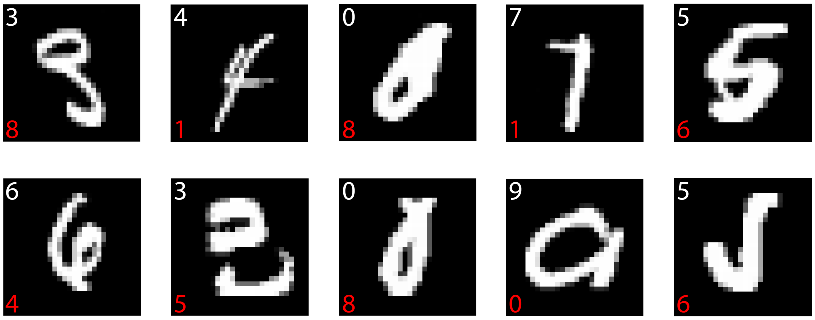

Supplementary Figure S8. Misclassified digits in the online MNIST classification task. The white digits at the top left corners are the true classes and the red digits at the bottom left are misclassified labels. Samples with unclear writings could be recognised incorrect digits of similar shape (i.e. 3 and 8, 9 and 0).

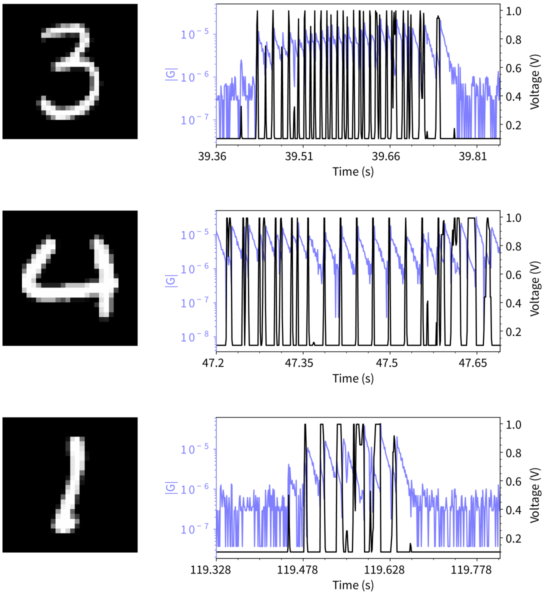

Supplementary Figure S9. Input voltage and network conductance for different MNIST digit inputs. Network conductance gradually decays after high intensity pixels are delivered, demonstrating the fading memory property.

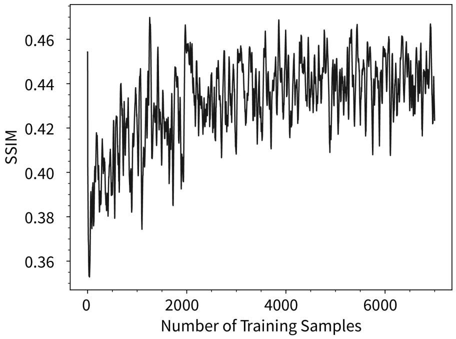

Supplementary Figure S10. SSIM as a function of number of training samples used to train the online reconstructor. As the image reconstructor learns from more samples, SSIM increases rapidly initially then more gradually. The SSIM reported in the main results is the value after training with 7000 samples. A memory window of length $L = 4$ is shown here as an example.

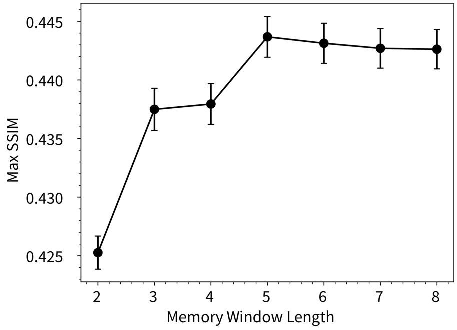

Supplementary Figure S11. Maximum SSIM averaged across all digits as a function of memory window length. Max SSIM improves with memory window length, as more features are used to train the online image reconstructor. Max SSIM plateaus after the length of the memory window is greater than 4 because the reconstructor is able to use half of the semi-repetitive sequence. Errorbars indicate the standard error of the mean across all digits for reconstructions.

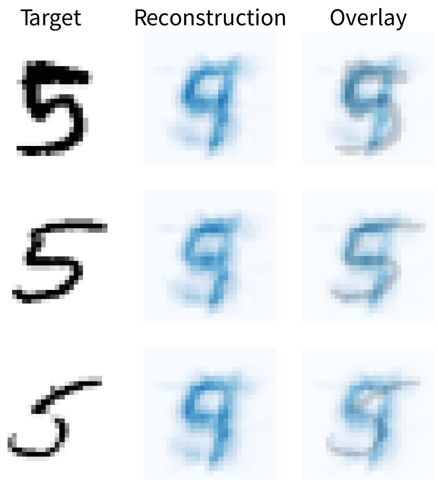

Supplementary Figure S12. Reconstruction of digit 5 in the sequence memory task. Reconstruction of digit 5 shows interference from digit 9, which shares the same memory features from the same sub-sequence 147. A memory window of length $L = 4$ is used here.

	Online (w/o active error correction)		Batch	
Readout data	784 x1	784 x 5	784 x 1	784 x 5
1,000 testing	91.0%	94.1%	92.2%	93.8%
10,000 testing	85.9%	90.5%	86.6%	91.4%

Supplementary Table 1. MNIST classification accuracies without active error correction in the online algorithm. Without error correction, accuracy of the online method is comparable to the batch method. For comparison, results are shown for 1,000 and 10,000 testing samples (for 50,000 training samples). Main results are for 10,000 testing samples. See Supplmentary Figure S7 for results comparing different electrode combinations and number of channels using 1,000 testing samples.

Supplementary Note 1 – Memristive switching in Ag_2Se nanowire networks

Threshold switching in Ag_2Se nanowires was first reported by Schoen et al. [5], who found that switching from low to high conducting states is concomitant with (although not caused by) a structural phase transition. In the low conducting (cubic α) phase, Ag^+ cations form an amorphous sublattice in which the high resistance may be attributed to localised trap states close to the conduction band edge. Under an electrical bias, a conducting Ag nanofilament forms across an individual nanowire. In Ag_2Se nanowire networks, Ag_2Se nanowires are synthesised in their orthorhombic β phase and volatile threshold switching is observed and attributed to Ag nano-filament formation at the cross-point junctions [6]. The memristive behaviour reported here, in particular the non-zero crossing in the $I-V$ curves (cf. Supplementary Figure S3), suggests that in addition to electro-chemical metallisation, other nanoscale processes may be operating at the cross-point junctions. Valov et al. [7] show that in redox-based memristive units, an electromotive force (effectively, a nanobattery) can be induced by strong spatial gradients in the distribution of Ag^+ ions and their mobilities. This results in a non-equilibrium diffusion potential that manifests as a non-zero $I-V$ crossing. Our findings are consistent with this picture, showing that the effect becomes increasingly noticeable under non-adiabatic conditions, when the nanowire network is driven under higher frequency AC. Similar frequency-dependent behaviour has also been observed in individual TiO_2 devices and attributed to memcapacitive and meminductive properties [8].

References

[1] Kuncic, Z. *et al.* *Emergent brain-like complexity from nanowire atomic switch networks: Towards neuromorphic synthetic intelligence*, 1–3 (2018).

[2] Kuncic, Z. *et al.* *Neuromorphic Information Processing with Nanowire Networks*, 1–5 (2020).

[3] Hochstetter, J. *et al.* Avalanches and edge-of-chaos learning in neuromorphic nanowire networks. *Nature Communications* **12** (1), 4008 (2021). https://doi.org/10.1038/s41467-021-24260-z .

[4] Zhu, R. *et al.* Information dynamics in neuromorphic nanowire networks. *Scientific Reports* **11** (1), 13047 (2021). https://doi.org/10.1038/s41598-021-92170-7 .

[5] Schoen, D. T., Xie, C. & Cui, Y. Electrical Switching and Phase Transformation in Silver Selenide Nanowires. *Journal of the American Chemical Society* **129** (14), 4116–4117 (2007). https://doi.org/10.1021/ja068365s .

[6] Kotooka, T. *et al.* Ag2Se Nanowire Network as an Effective In-Materio Reservoir Computing Device. Preprint (2021).

[7] Valov, I. *et al.* Nanobatteries in redox-based resistive switches require extension of memristor theory. *Nature Communications* **4** (1), 1771 (2013). https://doi.org/10.1038/ncomms2784 .

[8] Qingjiang, L. *et al.* Memory Impedance in TiO2 based Metal-Insulator-Metal Devices. *Scientific Reports* **4** (1), 4522 (2014). https://doi.org/10.1038/srep04522 .
